# Immediate Breast Tissue Expander-Implant Reconstruction With Inferolateral AlloDerm Hammock and Postoperative Radiation: A Preliminary Report

**Published:** 2009-05-15

**Authors:** Karl H. Breuing, Amy S. Colwell

**Affiliations:** Department of Surgery, Division of Plastic Surgery, ^a^Brigham and Women's Hospital; ^b^ Mass General Hospital, Harvard Medical School, Boston, MA 02115.

## Abstract

**Disclosures:** K.H.B. is on the speaker's bureau for LifeCell. **Objective:** To preserve the mastectomy skin envelope in select patients destined to receive radiation following mastectomy, we performed immediate tissue expander-implant reconstruction with a subpectoral tissue expander and an inferolateral AlloDerm hammock for complete implant coverage. We hypothesized that the AlloDerm hammock may allow greater intraoperative volume expansion and potentially avoid the need for an autologous construct. **Methods:** Tissue expanders were filled to 75%–85% capacity intraoperatively and 85%–100% prior to radiation therapy. This allowed for maximum preservation of the mastectomy skin envelope prior to radiation therapy and provided a sizable breast mound immediately following mastectomy. Histology of irradiated and nonirradiated capsules was compared. **Results:** Five patients aged 29–51 years had immediate implant (1) or expander-implant (4) breast reconstruction followed by postreconstruction radiation 2–6 months following the procedure. Patients were followed for 2.5–5.5 years following implant reconstruction and 2–5 years following radiation. No capsular contracture or implant loss was observed in any patient. No patients required or requested autologous reconstruction following radiation and all currently have silicone implants. Capsular biopsies from radiated and nonradiated implants showed identical collagen architecture on histology, confirming clinical observations. **Conclusion:** Tissue expander-implant breast reconstruction following mastectomy preserves the skin envelope in patients who receive postmastectomy radiation. Further investigation is warranted to determine whether complete implant coverage with the pectoralis muscle and AlloDerm hammock mitigates the deleterious effects of radiation.

Immediate breast reconstruction with implants or expanders is the most common method of reconstructing the breast following mastectomy.^1^ Implant reconstruction allows young, otherwise healthy women an expedient return to work and their active lifestyles. In addition, autologous reconstructive options are preserved for recurrent breast cancer or implant failure. The recent introduction of an inferolateral cadaveric dermis (AlloDerm, Lifecell, Branchburg, NJ) hammock for immediate reconstruction allows improved positioning of the implant or expander in the postmastectomy pocket and thus a superior aesthetic results.^2^–^5^ In addition, the pectoralis muscle-AlloDerm pocket may protect the mastectomy skin by decreasing the gravitational force that would otherwise be transmitted to the lower pole envelope when the patient is standing or sitting.

The role of implant breast reconstruction in patients who have had radiation or who will undergo postmastectomy/postreconstruction radiation is controversial. A high incidence of implant loss, infection, and capsular contracture has been reported in this population.^6^–^8^ For these reasons, breast reconstruction is deferred by most surgeons if radiation therapy is part of the intended postmastectomy treatment plan. Following radiation, the skin envelope contracts and stiffens. Therefore, implant reconstruction is deemed not feasible and delayed reconstruction is performed with autologous tissue. Although autologous tissue can match the contralateral breast in contour, the skin island is invariably different in color and is surrounded by a scar, which may negatively impact the overall aesthetic outcome.

To maximize the amount of postmastectomy skin available for reconstruction after completion of radiation therapy, and potentially avoid the need for an autologous skin island, we placed an implant or tissue expander into a retropectoral pocket and covered the inferolateral implant surface with AlloDerm as described for immediate implant reconstruction.^3^ Tissue expanders were filled to 75%–85% capacity intraoperatively and to 85%–100% prior to radiation. We report initial results of 5 patients who underwent immediate reconstruction with implants or expanders and an inferolateral AlloDerm hammock followed by postoperative radiation therapy. No capsular contracture or implant loss has been observed in their 1- to 4-year follow-up.

## METHODS

This retrospective study was performed according to institutional guidelines. Chart review was performed on patients who had immediate breast reconstruction with implants or expanders followed by postreconstruction radiation.

In all cases of expander-implant reconstruction, the expanders were filled with injectable saline up to 85%–100% of their capacity during the initial reconstruction as determined by the amount of mastectomy skin envelope available for complete tension-free implant coverage. All expanders were placed into a retropectoral pocket, and AlloDerm was used to cover and suspend the portion of the implant not covered by muscle as previously described.^3^

Histology was performed on capsule biopsies 1 year after completion of radiation therapy in one patient with bilateral tissue expander-implant reconstructions who received radiation on one side.

## RESULTS

**Figure 1:**
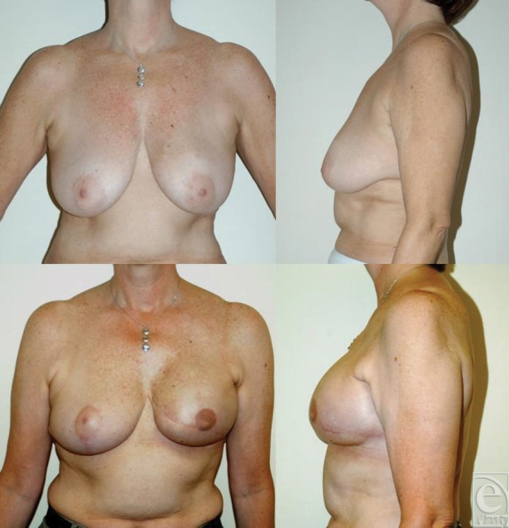
Immediate left-sided tissue expander-implant reconstruction following mastectomy and postoperative radiation. This 50-year-old woman received radiation 3 months after immediate tissue expander reconstruction. She subsequently underwent implant exchange and nipple construction and is shown 1 year following radiation.

**Figure 2:**
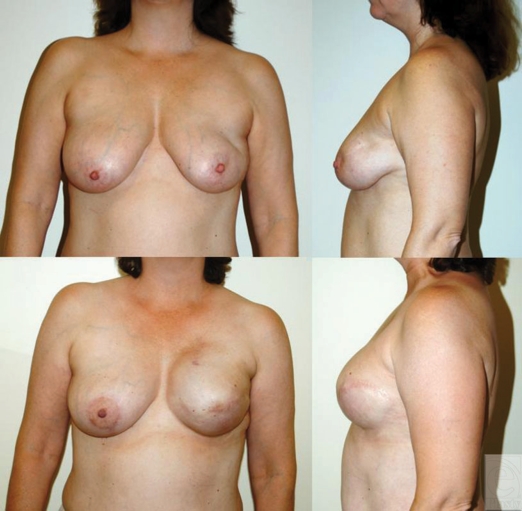
Immediate left-sided tissue expander-implant reconstruction following mastectomy and postoperative radiation. This 46-year-old woman received radiation 4 months after immediate tissue expander reconstruction. She is shown here at her 1-year follow-up.

**Figure 3:**
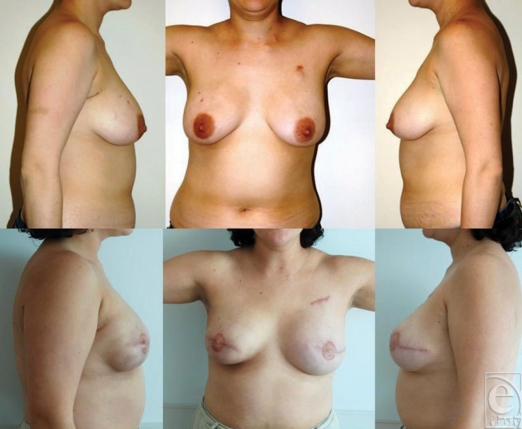
Immediate bilateral tissue expander-implant reconstruction following bilateral mastectomy and postoperative radiation to the *right* side. This patient had bilateral immediate tissue expander placement and then subsequently had radiation to the right side only. She then had an implant exchange and is shown here 1 year after radiation and after nipple areolar reconstruction and tattoo. The 2 sides are nearly indistinguishable.

**Figure 4:**
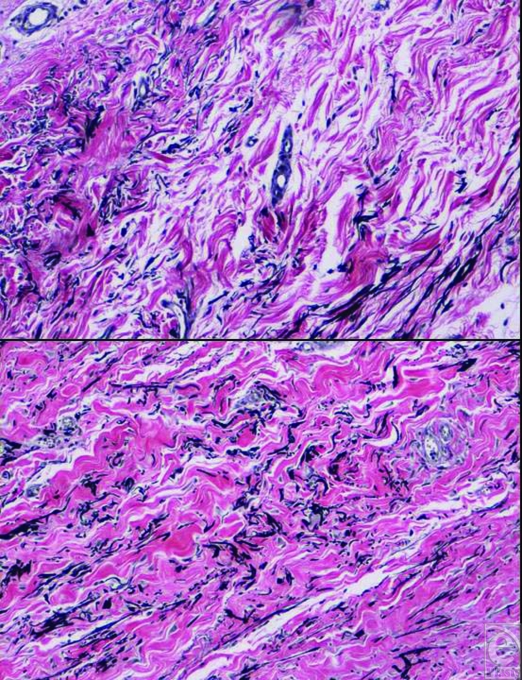
Histology of tissue expander-AlloDerm capsule at the time of bilateral implant exchange. The upper photo shows the nonradiated left breast capsule and the bottom photo shows radiated capsule. The two capsules are indistinguishable (Verhoff stain).

Five patients aged 29–51 years had immediate implant (1) or expander-implant (4) breast reconstruction followed by postreconstruction radiation (Table 1). Radiation was given 2–6 months following the procedure. Patients were followed for 2.5–5.5 years following implant reconstruction and 2–5 years following radiation (Figs 1–3). Histology of the implant capsules in a patient with bilateral reconstruction and radiation on one side showed indistinguishable collagen architecture 1 year after radiation therapy (Fig 4). Complications included one small wound dehiscence following implant reconstruction but before radiation. This was treated with local excision and closure. Neither infection, capsular contracture, or implant extrusion nor loss of implant was observed in any patient.

## DISCUSSION

Immediate breast reconstruction with an implant or expander-implant and an inferolateral AlloDerm hammock offers a cosmetically acceptable reconstruction option for young, active women or those who desire preservation of autologous tissue and a rapid recovery from surgery. Radiation is a formidable foe, and complication rates of radiation combined with implant reconstruction have often deterred surgeons from offering this option to breast cancer patients.

**Figure 5:**
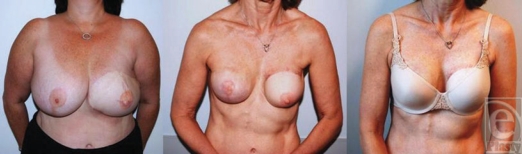
Delayed breast reconstruction with TRAM flap in 2 patients after left-sided mastectomy and XRT. Despite good match in volume and shape, the aesthetic results are compromised because of visibility of TRAM skin island (scar and color mismatch) which can extend beyond the border of the bra.

Delayed reconstruction with autologous tissue has been the mainstay of breast reconstruction in the setting of postoperative radiation therapy. The use of autologous tissue for delayed reconstruction can lead to a successful outcome; however, there are certain limitations. A subset of women lacks suitable autologous donor sites. Another subset simply refuses autologous options secondary to their active lifestyle or the associated morbidity of the procedures. Aesthetically, the contour of autologous reconstructions is generally quite good. However, the color mismatch of autologous skin surrounded by irradiated mastectomy skin can detract from the overall result. In addition, if the construct is placed too superior or medial, the scars may limit choice of clothing in some cases (Fig 5). In this report, we describe 5 patients who underwent immediate implant or tissue expander/implant reconstruction with an AlloDerm hammock followed by radiation therapy. No patient lost her implant or developed capsular contracture. This preliminary observation has served as the impetus for a larger institutional review board (IRB)–approved study currently underway.

AlloDerm is being used with increasing frequency in aesthetic and reconstructive surgery for rhinoplasty, hand surgery, lip augmentation, chest wall reconstruction, abdominal wall reconstruction, mastopexy, and nipple reconstruction. Its soft, pliable consistency makes it easy to work with, and its safety has been demonstrated by clinical experience over the past 10 years. However, there are few articles that address the effects of radiation on AlloDerm. In a rat model, Dubin et al^9^ demonstrated that graft thickness and neovascularization of AlloDerm were not adversely affected by a field that had received external beam radiation (EBR). Ibrahim et al^10^ implanted AlloDerm into rat hind legs and subsequently delivered 20 Gy of EBR. In this model, EBR hindered recellularization of the AlloDerm in the early posttreatment period, but graft thickness, recellularization, and graft survival were not adversely affected at 12 weeks.^10^

In our experience over the past 6 years, the addition of an AlloDerm hammock to breast implant reconstruction has offered superior aesthetic results by allowing precise implant positioning in the mastectomy pocket.^3^ Furthermore, capsular contracture in these patients is remarkably diminished. Although implant reconstruction has been performed successfully in patients before or after radiation therapy, a high rate of contracture has been reported and up to 50% of these patients may ultimately require an autologous construct.^6^,^7^ For this reason, prior to our experience with AlloDerm, we typically avoided implant reconstruction in combination with radiation.

Serendipitously, we performed immediate bilateral reconstruction with silicone implants and AlloDerm slings in a patient who was not scheduled for radiation as part of her treatment. Postoperatively, we were informed that there had been a change of plans and the patient received for breast irradiation. Much to our surprise, she did not develop capsular contracture in the following 5 years.

On the basis of this positive experience and the desire to preserve the mastectomy skin envelope, we subsequently performed immediate tissue-expander reconstruction with a subpectoral tissue expander and an inferolateral AlloDerm hammock in 4 select patients scheduled for postmastectomy radiation. Tissue expanders were filled approximately 75%–85% capacity intraoperatively and 85%–100% capacity prior to radiation. Filling the tissue expander to 85%–100% capacity prior to radiation therapy allows maximum preservation of the mastectomy skin envelope as little expansion can typically be achieved following radiation. In our patients, good mastectomy skin envelopes and the off-loading of mechanical stress by the pectoralis-AlloDerm pocket provided an ability to fill tissue expanders to 75%–85% capacity and provided a sizable breast mound immediately following mastectomy. This can be very important as radiation oncologists typically prefer to start radiation 6 weeks following mastectomy if no chemotherapy is needed or if it was received preoperatively. This limits the plastic surgeon's ability to expand the skin envelope prior to radiation.

Radiation therapy was typically started 6 weeks after mastectomy and reconstruction. We monitored all patients during their radiation therapy in 2-week intervals and did not observe any skin breakdown other than typical radiation-induced skin changes that were treated topically. As anticipated, no patient developed capsular contracture after completion of radiation therapy. To confirm our clinical observations, we performed capsular biopsies on a patient who had bilateral tissue expander reconstruction followed by radiation on one side (Fig 4). As expected, the collagen architecture on histology was identical. The shape of the reconstructed breast mounds remained aesthetically pleasing and the skin quality and color was comparable to the contralateral native breasts. It was therefore no surprise that all patients preferred implant exchange instead of autologous reconstruction at 6 months following radiation. In subsequent follow-up, all patients remained free from capsular contracture. Symmetry operations on the contralateral native breast were performed as needed.

On the basis of these positive results, we have been working closely with our radiation oncology department to design and implement an IRB-approved protocol to perform a larger study to prospectively evaluate the influence of postoperative radiation on implant-AlloDerm constructs. Our radiation oncologists limited the size of the current study as they believe immediate tissue expander and even autologous tissue reconstruction following left-sided mastectomy might prevent adequate delivery of radiation and necessitate an excessive cardiac radiation dose. They have since determined that most women with implants can be sufficiently treated, and that a preoperative chest computed tomography simulating pre-XRT mapping can help identify this small subgroup of patients who are not good candidates for preradiation implant or autologous reconstruction based on their chest wall dimensions.

In this report, we describe our favorable experience with postoperative radiation in 5 patients with expander-implant reconstruction and AlloDerm hammock. If our initial observations are substantiated, the expander-implant-AlloDerm construct may offer a reconstructive alternative for women destined for radiation who lack adequate donor sites or who wish to avoid the morbidity of autologous reconstruction.

## Figures and Tables

**Table 1:** Patient characteristics

	Age, y	Procedure	Chemotherapy	XRT^a^	F/u after XRT	F/u since implant or expander	Complications
1	51	Immediate Implant/AlloDerm	No	Postoperation	5 y 3 mo	5 y 6 mo	Small wound dehiscence before XRT– closure under local
2	29	Immediate Expander/AlloDerm	Yes	Postoperation	2 y	2 y 6 mo	
3	50	Immediate Expander/AlloDerm	Yes	Postoperation	2 y 7 mo	2 y 10 mo	
4	38	Immediate Expander/AlloDerm	Yes	Postoperation	2 y 8 mo	3 y	
5	46	Immediate Expander/AlloDerm	Yes	Postoperation	2 y 8 mo	3 y 3 mo

^a^XRT indicates radiation.
